# Supplementary material for: Concordant transcriptional and morphological remodeling revealed by in vivo Perturb-CLEAR
Source: bioRxiv. 2026 Apr 8:2026.04.06.716787. Preprint. [Version 1] doi: 10.64898/2026.04.06.716787 (PMC13081986; doi:10.64898/2026.04.06.716787)

Extended Data Figure 1

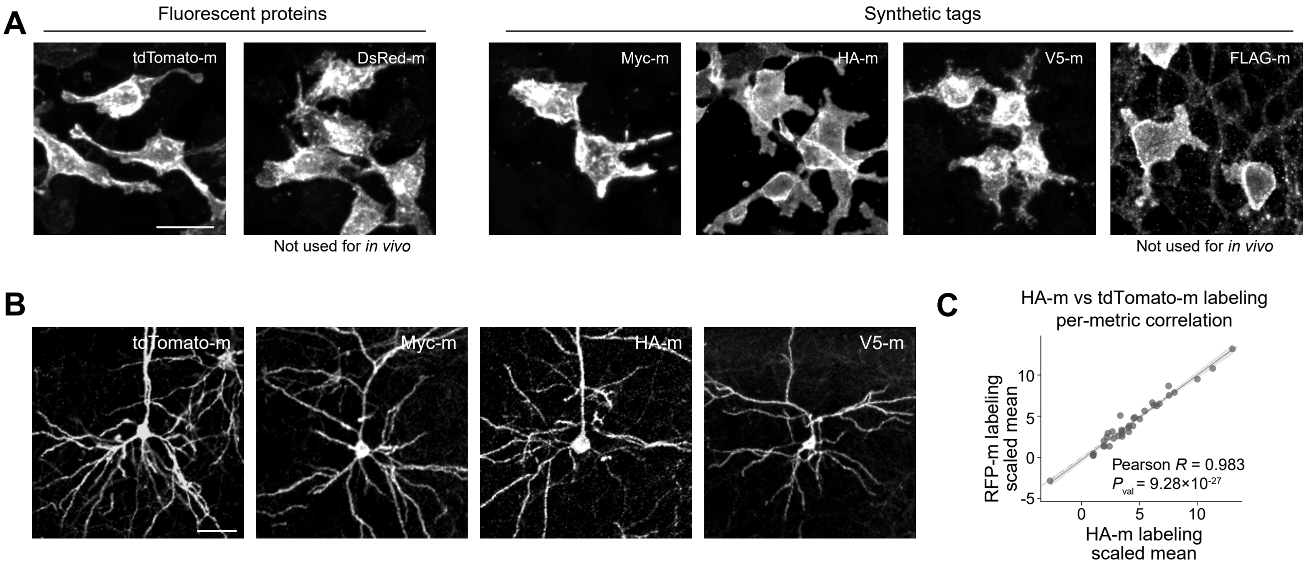

Extended Data Figure 2

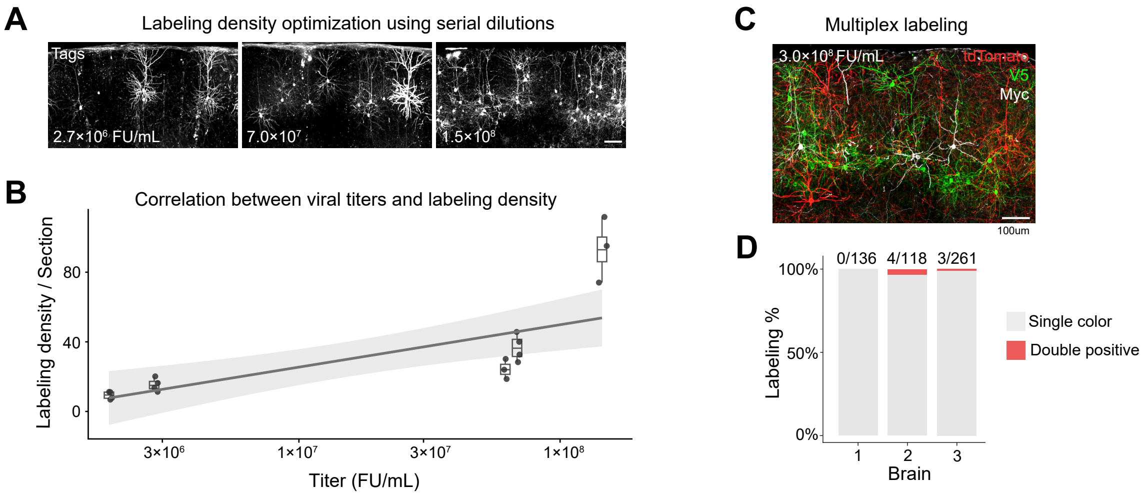

Extended Data Figure 3

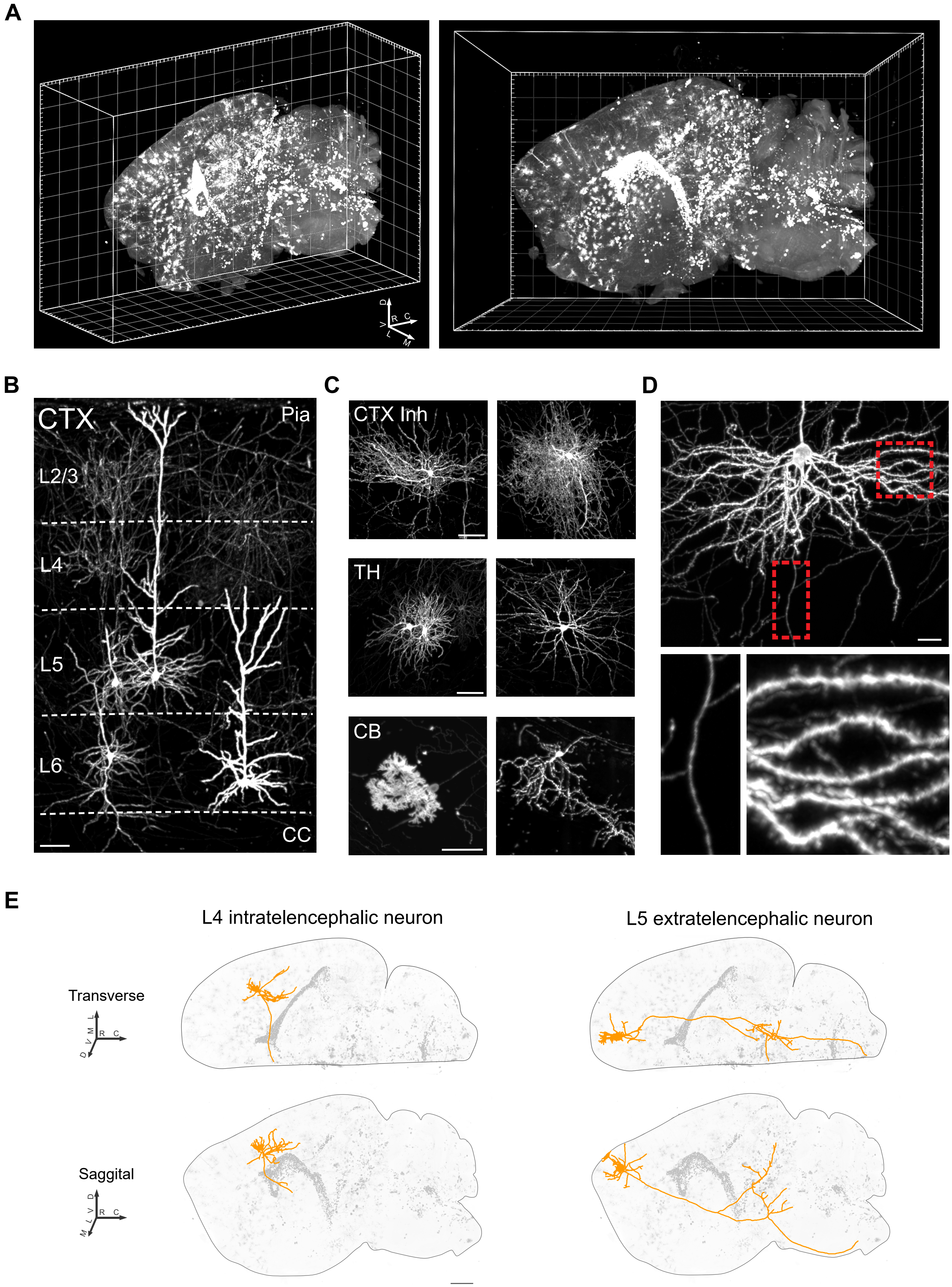

Extended Data Figure 4

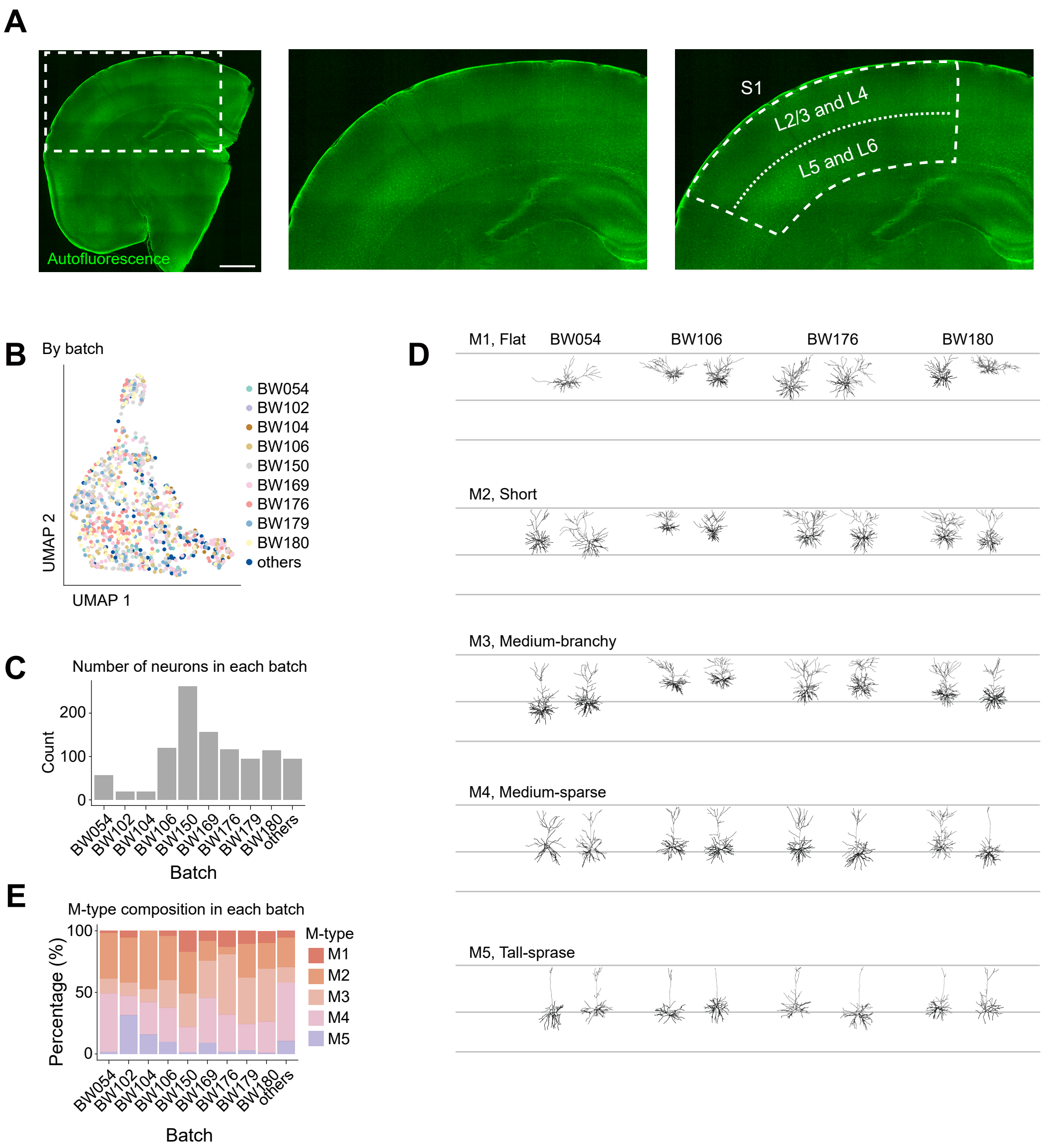

Extended Data Figure 5

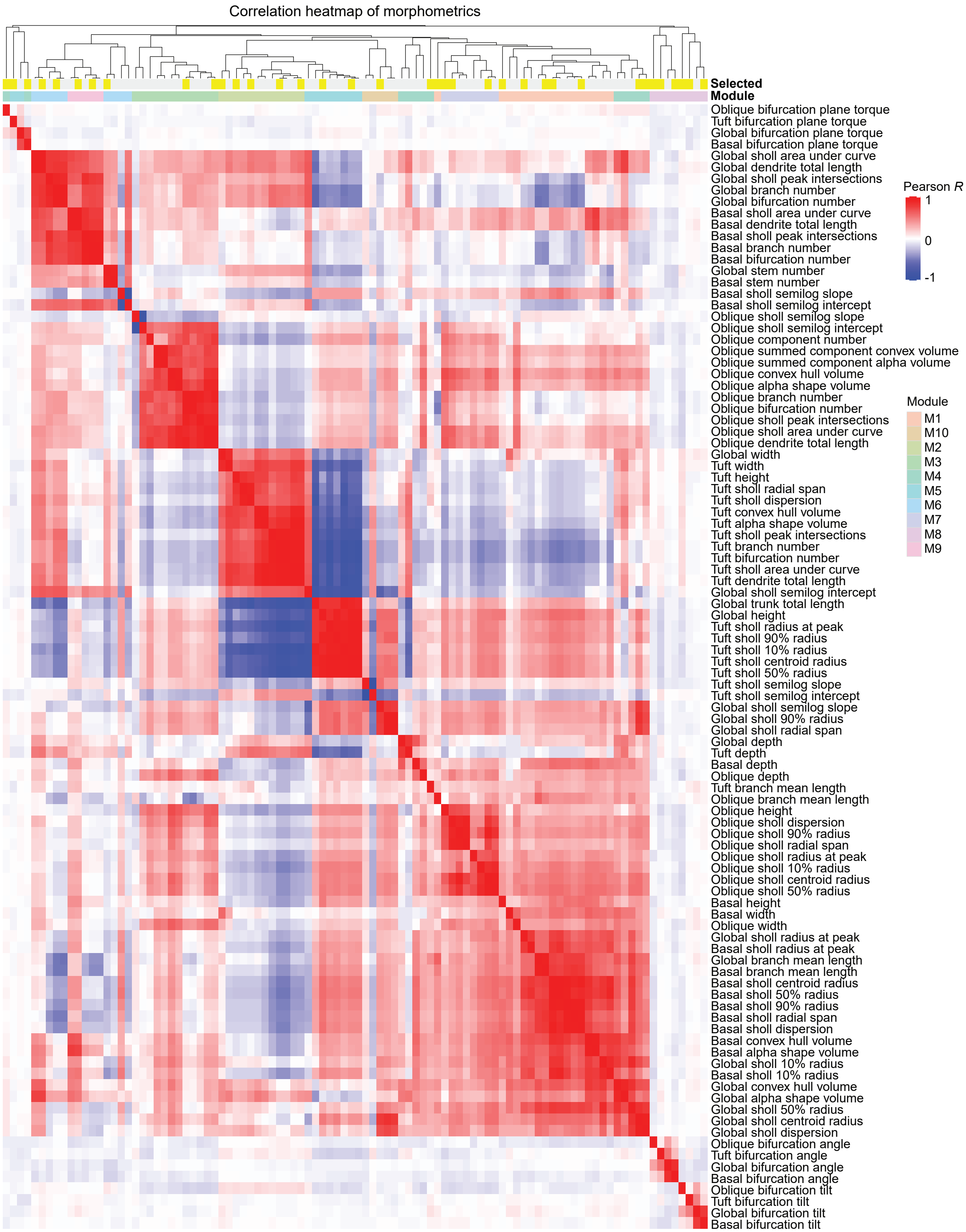

Extended Data Figure 6

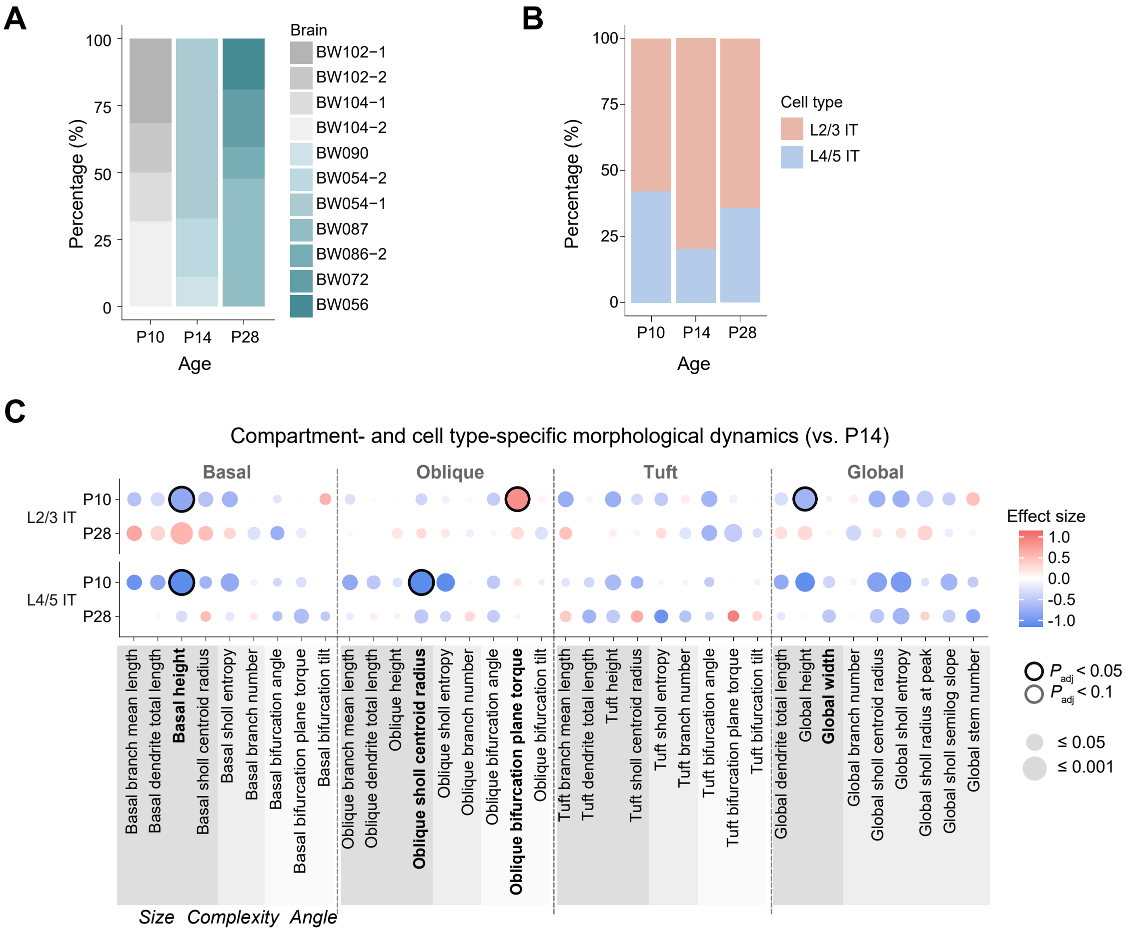

Extended Data Figure 7

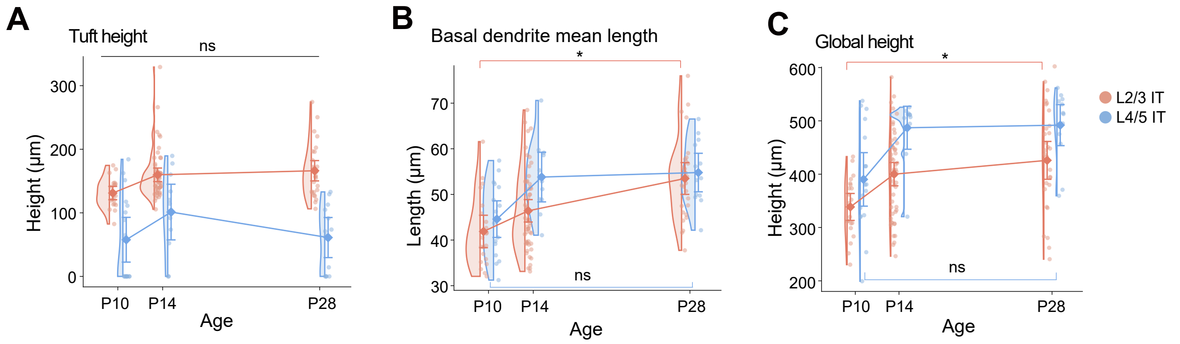

Extended Data Figure 8

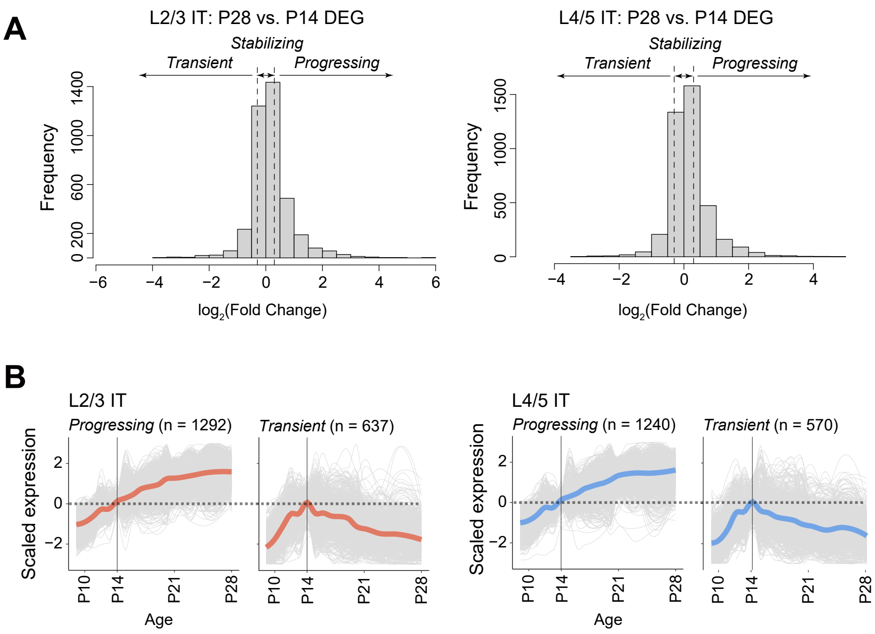

Extended Data Figure 9

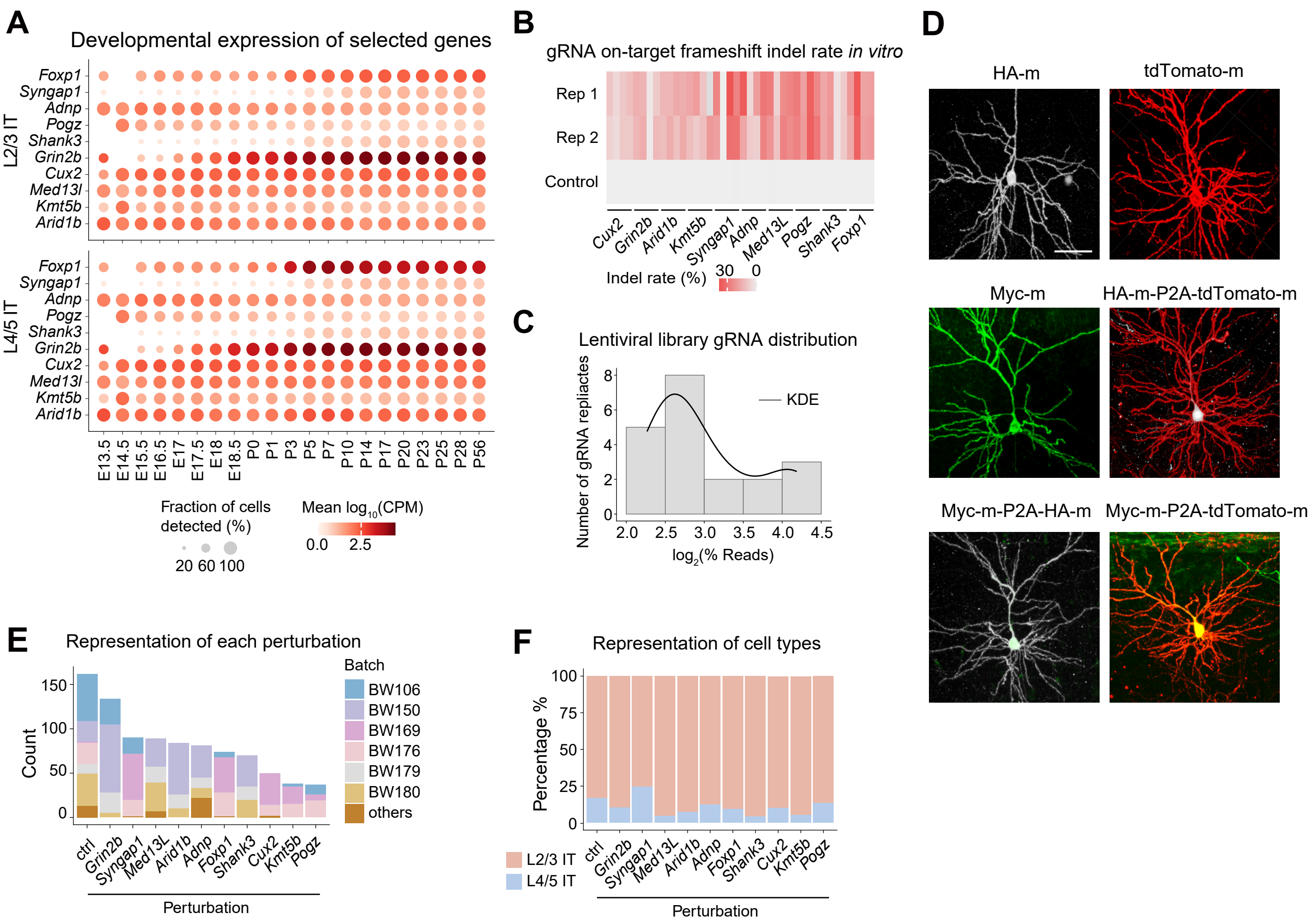

Extended Data Figure 10

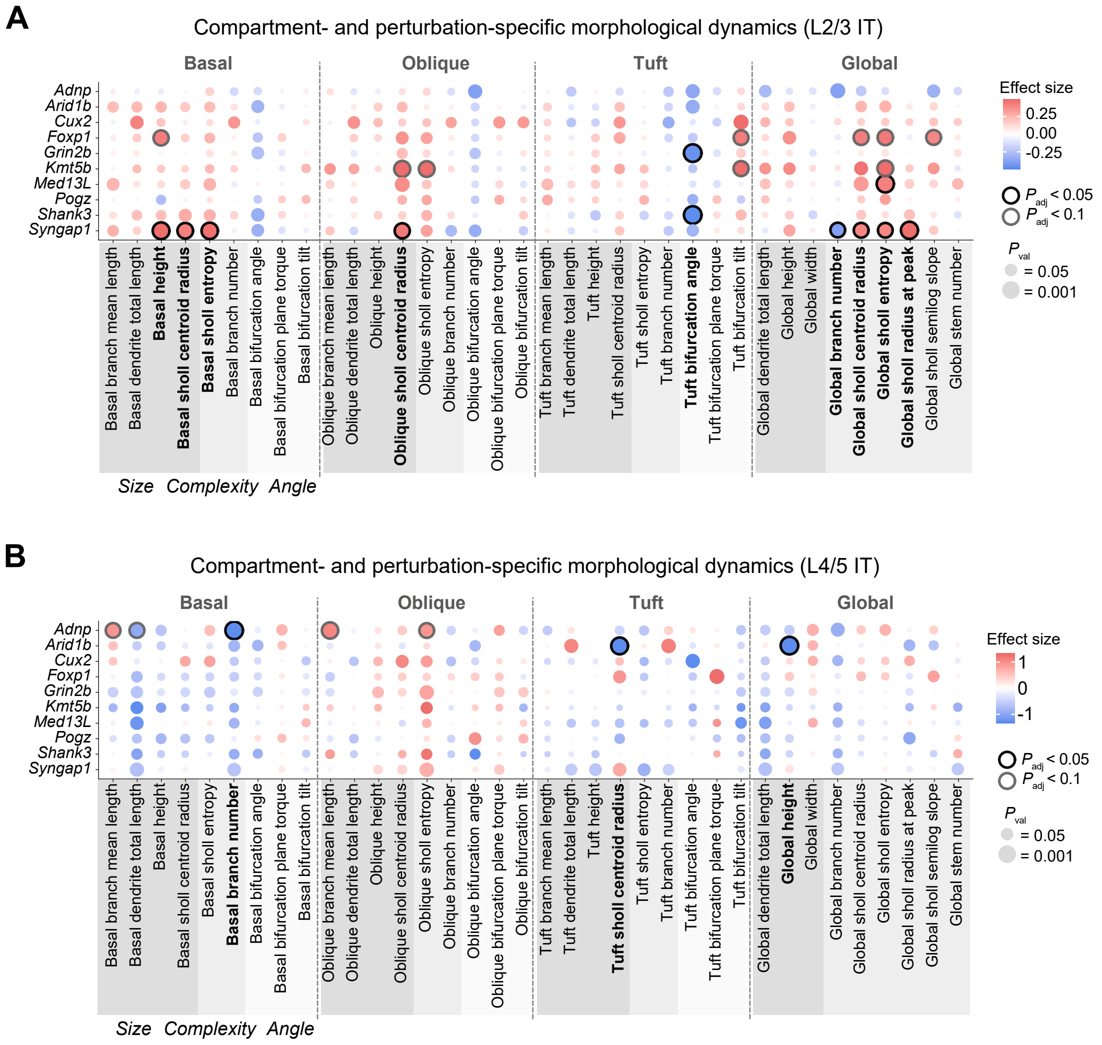

Extended Data Figure 11

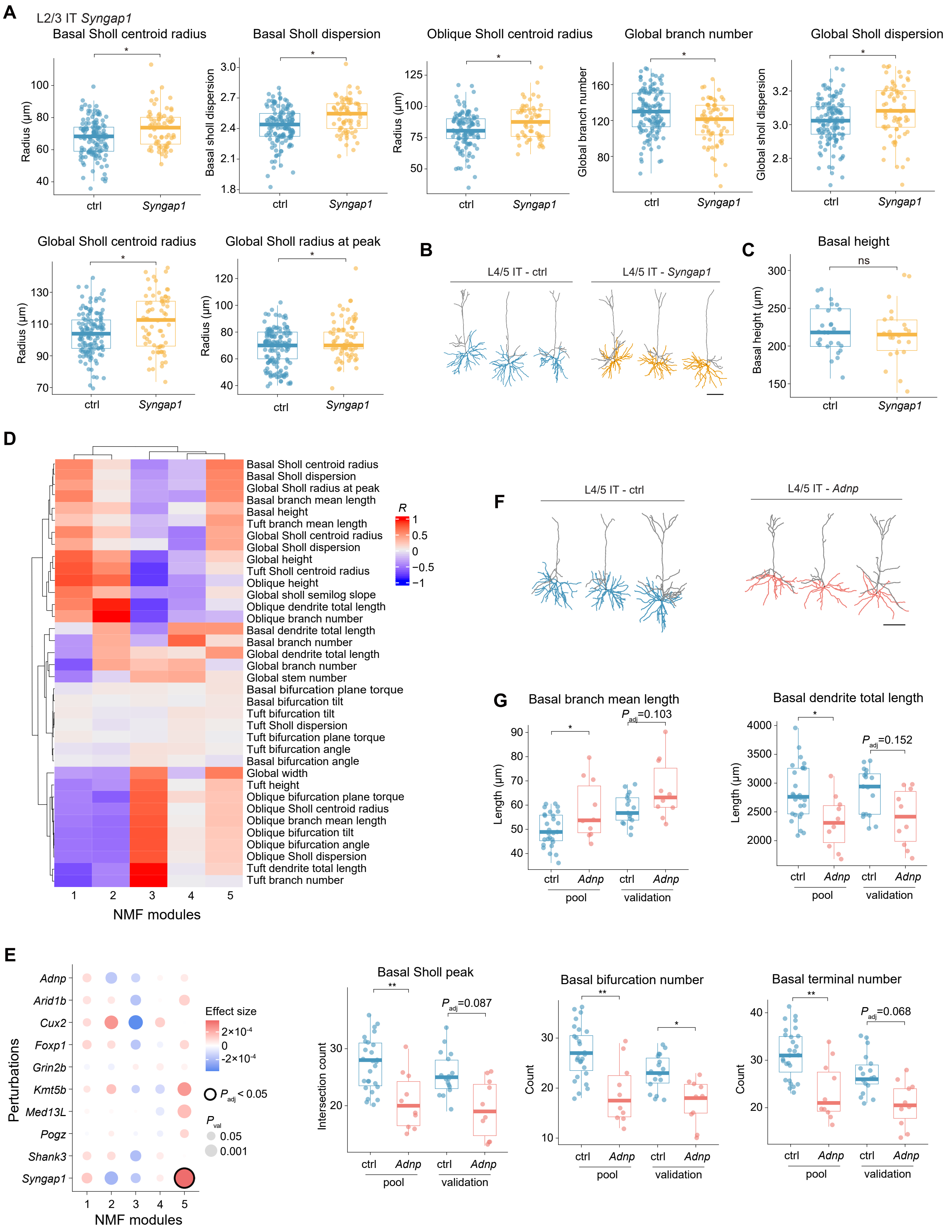

Extended Data Figure 12

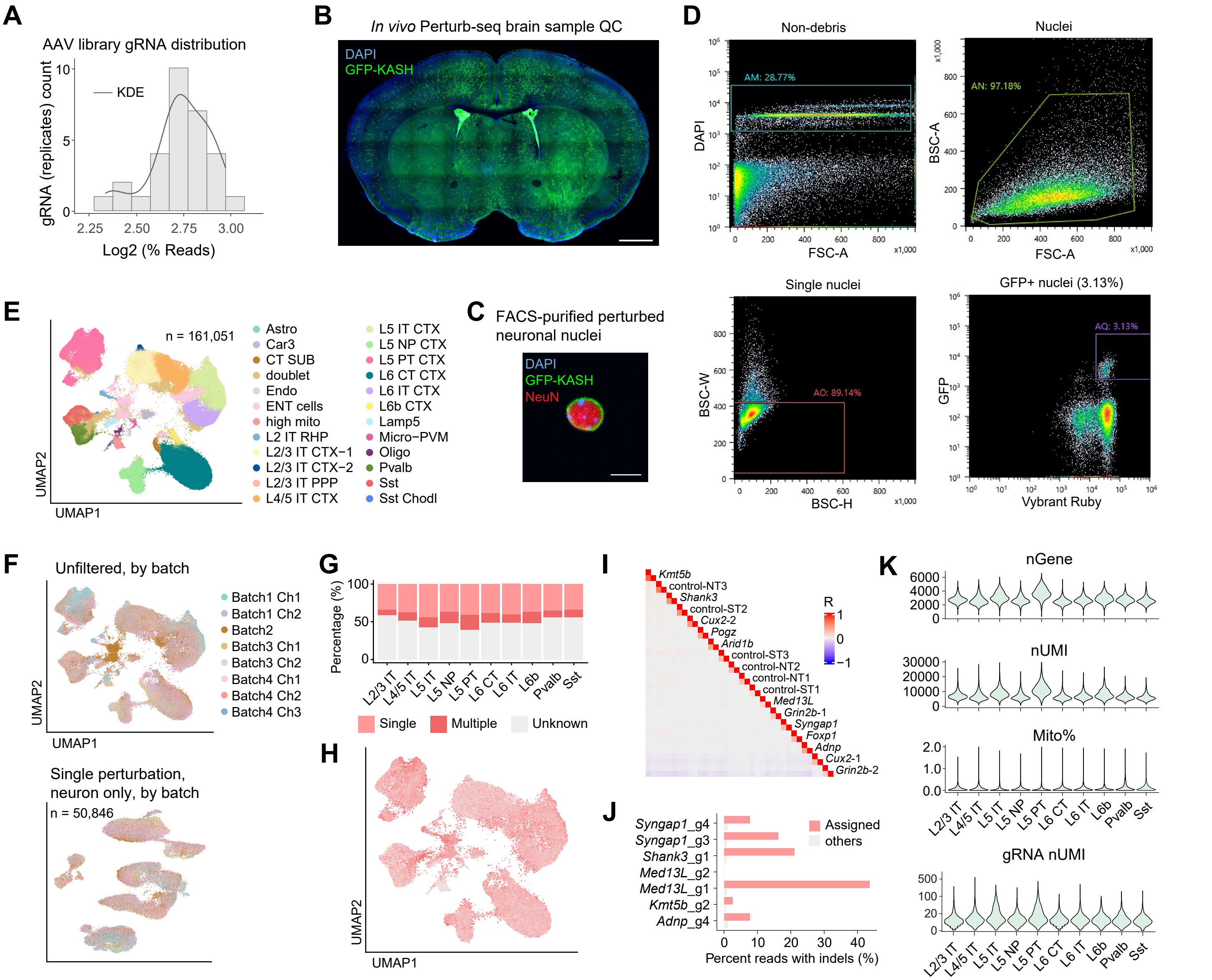

Extended Data Figure 13

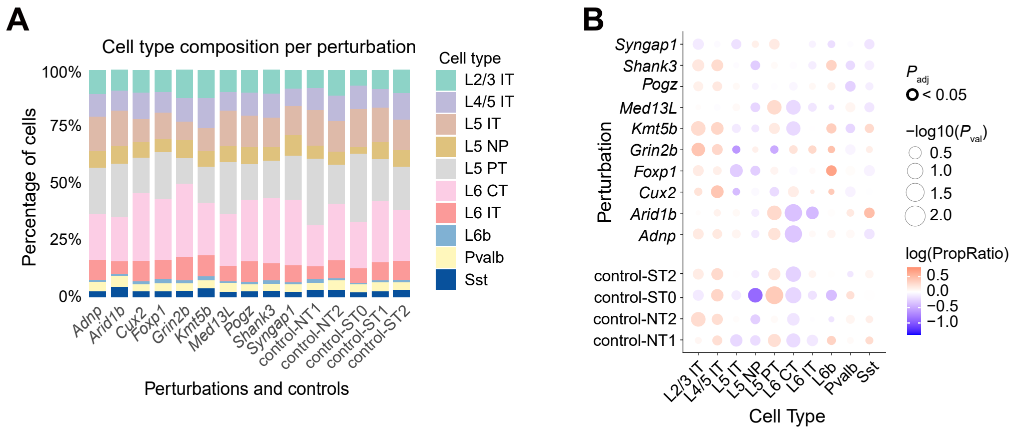

Extended Data Figure 14

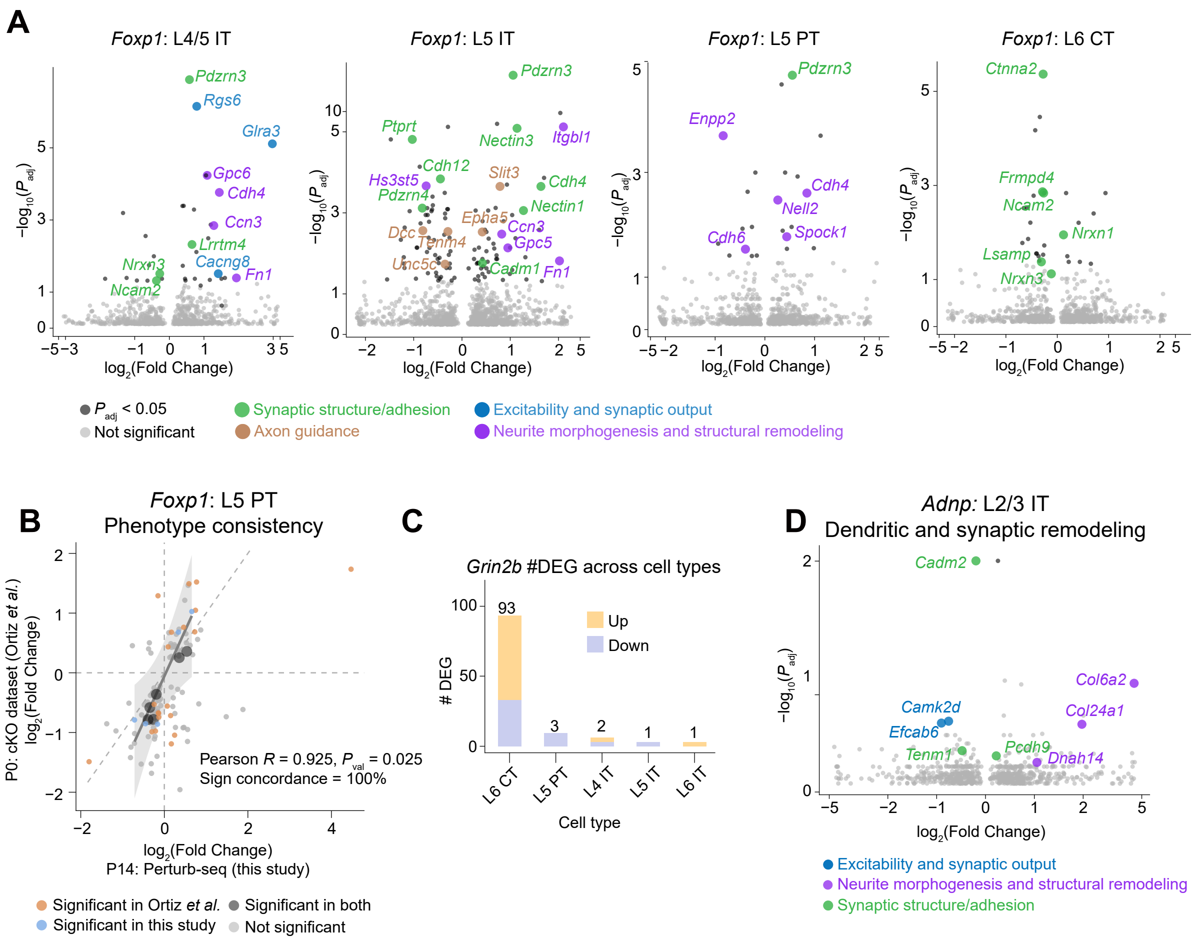

Extended Data Figure 15

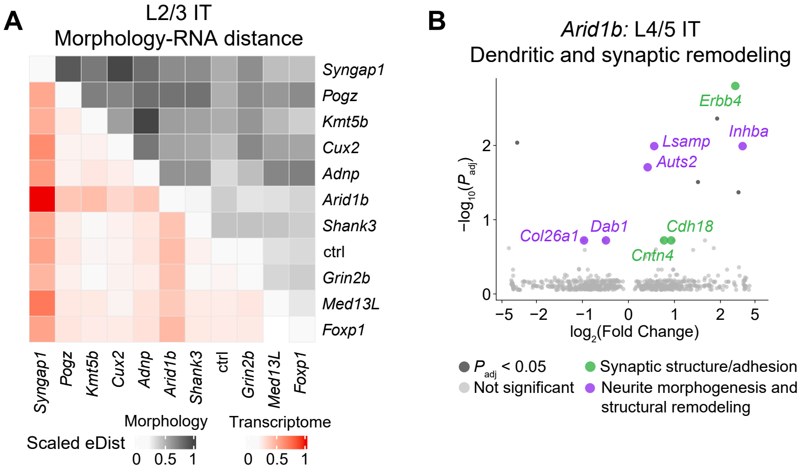

Supplement: Supplement 2 — Extended Data Fig. 1. In vitro and in vivo screen of labeling reporters with consistent and high performance cytoarchitectural readouts. A. Immunostaining showing membrane-tethered reporters labeling in transfected HEK293T cells. tdTomato-m: membrane-tethered tdTomato, DsRed-m: membrane-tethered DsRed. Myc-m: membrane-tethered smFP.Myc, HA-m: membrane-tethered smFP.HA, V5-m: membrane-tethered smFP.V5, FLAG-m: membrane-tethered smFP.FLAG. tdTomato-m, Myc-m, HA-m, and V5-m were used for in vivo test. Scale bar: 20 μm. B. Immunostaining showing membrane-tethered reporters labeling in transduced neurons in vivo. Scale bar: 50 μm. C. Correlation plot showing high consistency between the SD-scaled means of MetricSet1 derived from Perturb-CLEAR-labeled neurons using different tags. A fitted linear regression line is shown with 95% confidence band. Dashed line indicates y = x. tdTomato-m and HA-m-labeled wild-type P10 L4/5 IT neurons are used as examples, tdTomato-m: n = 7 from N = 3 brains; HA-m: n = 9 from N = 4 brains. Extended Data Fig. 2. Viral titer optimization allows appropriate labeling density for dendritic mapping and reliable perturbation identity assignment. A. Immunostaining showing the labeling density in somatosensory cortex across titers (2.7×10^6^– 1.5×10^8^ FU/mL). Maximum intensity projections from 400 μm thick slab from whole-mount images. Scale bar: 200 μm. B. Correlation between injection titers and labeling density of Perturb-CLEAR brain samples. Titer is plotted on a log10 scale, whereas density is shown on the original scale. The interquartile range (IQR) with median (center line) is shown, with whiskers extending to 1.5× IQR; overlaid jittered points indicate individual brains. A fitted linear regression line (solid) is shown with 95% confidence band. n > 3 brains for each condition. FU: functional unit. C. Immunostaining of somatosensory cortex from a multiplex labeled brain sample (mixed lentiviral vectors with tdTomato-m, V5-m, and Myc-m, 3.0×10^8^ F [file media-2.pdf]
